# Crowdsourcing and Minority Languages: The Case of Galician Inflected Infinitives^1^

**DOI:** 10.3389/fpsyg.2019.01157

**Published:** 2019-06-25

**Authors:** Michelle Sheehan, Martin Schäfer, Maria Carmen Parafita Couto

**Affiliations:** ^1^School of Humanities and Social Sciences, Anglia Ruskin University, Cambridge, United Kingdom; ^2^SFB 833: The Construction of Meaning, University of Tübingen, Tübingen, Germany; ^3^Center for Linguistics, Leiden University, Leiden, Netherlands

**Keywords:** inflected infinitives, phases, finiteness, crowdsourcing, sociolinguistics, audio-questionnaire, control

## Abstract

Results from  a crowdsourced audio questionnaire show that inflected infinitives in Galician are acceptable in a broad range of contexts, different from those described for European Portuguese. Crucially, inflected infinitives with referential subjects are widely accepted only inside strong islands in Galician (complements of nouns, adjunct clauses). They are widely rejected in non-islands, notably in the complements of epistemic/factive verbs, in contrast with Portuguese and older varieties of Galician (Gondar, 1978; Raposo, 1987). Statistical analysis shows, however, that, in the complements of epistemic/factive (and desiderative) verbs, inflected infinitives are significantly more acceptable in instances of control, whether partial or exhaustive. In fact, there is no significant difference between these two types of control in Galician, unlike in Portuguese, where inflection is generally better in instances of partial control and is not acceptable in instances of exhaustive local subject control (Modesto, 2010; Sheehan, 2018a). We propose an analysis of this pattern in terms of phase theory. The inflectional domain of non-finite clauses remains visible to the thematic domain of the next clause up, according to the less strict version of the Phase Impenetrability Condition (Chomsky, 2001), allowing control to take place. Pronouns/or pronominal inflections in the inflectional domain of visible non-finite clauses therefore get controlled. In islands, however, material in the inflectional domain remains free/referential. Despite this basic pattern, the data are characterized by substantial interspeaker variation. Statistical analysis shows that gender, urban/rural birthplace and mother tongue are all significant factors in this variation, while age and region of birth are not. Most notably, urban-born male bilinguals with Spanish as their mother tongue consistently rate all sentences higher on the Likert scale. Overall, the results show that crowdsourcing can lead to empirically robust syntactic descriptions of minority languages which are likely to be subject to substantial sociolinguistic variation and where judgments from a single social group may be misrepresentative of the general picture. The study also highlights, however, the challenges associated with using crowdsourced audio-questionnaires of this kind and the need for statistical analysis of results to control for substantial amounts of variation.

## Introduction

This article argues that crowdsourced audio-questionnaires are well-suited for the investigation of the syntactic properties of minority languages. We illustrate this in relation to Galician, a minority language spoken mainly in Galicia in north-west Spain with a total of 2,372,000 speakers (Simons and Fennig, 2018), all of whom are estimated to be bilingual in Spanish. More specifically, we report on speakers’ intuitions regarding inflected infinitives in Galician, based on an online audio-questionnaire using a five-point Likert scale acceptability judgment task.

Galician, like Portuguese, Mirandese, Old Leonese and some Italian dialects (Sardinian, Old Neopolitan) has both inflected and uninflected infinitives (Longa, 1994; Ledgeway, 1998; Scida, 2004). In Galician, which unlike many varieties of Portuguese, preserves the 2PL informal pronoun *vós* and its associated inflection, inflected infinitives are morphologically marked in all person/number combinations except 1SG/3SG, with the latter being homophonous with uninflected infinitives. The following example illustrates this for the irregular verb *ser* ‘to be’:


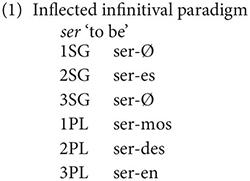


This verb form is mainly limited to subordinate clauses, though not exclusively, and it differs from the subjunctive in being banned from finite clauses. Although the inflected infinitive is a salient feature of Galician, and a property not shared with Spanish, its syntactic properties have not been widely studied (but see Gondar, 1978; Longa, 1994; Jansegers and Vanderschueren, 2010). Crowdsourcing via modern technology offers the perfect chance to collect acceptability judgments from large numbers of speakers across the region. The main aim of our survey is thus to use this technique to establish exactly where Galician speakers accept the inflected infinitive in the spoken language, to see to what extent acceptability is conditioned by social variables and to discuss the implications of our findings for syntactic theory. We use an audio questionnaire for this purpose in an attempt to tap into speakers’ intuitions about spoken, rather than written Galician, which has been claimed to make greater use of inflected infinitives (see Gondar, 1978 and below).

The remainder of this article is structured as follows. Section 2 gives some background on the Galician language and the morphology and syntax of inflected infinitives, based largely on Gondar (1978). It also briefly presents the theoretical issues for which these data are potentially important. Section 3 presents the materials and methods of the present survey. Section 4 provides a statistical analysis of the results of the survey. Section 5 discusses the implications of these results for syntactic theory. Finally, section 6 briefly discusses the benefits and drawbacks of crowdsourcing for the syntactic study of minority languages, drawing on the insights of this study. Finally, section 7 concludes.

### Background on Galician Inflected Infinitives and Control

#### Galician Inflected Infinitives

Galician became an official language in Galicia in 1978 and moved quickly through the process of written standardization (Santamarina Fernández, 1994; Kabatek, 1997; Ramallo and Rei-Doval, 2015). Many grammatical aspects of the language, including the use of inflected infinitives are yet to be officially documented, however, as the *Real Academia Galega* (Royal Academy of the Galician Language), established in 1906, has not yet published an official Galician grammar, leading to the lack of a clear normative standard (Álvarez et al., 2004). In fact, there has been very little descriptive work on the Galician inflected infinitive and little consideration of its relevance for syntactic theory, despite the fact that it has long been claimed to differ from its much better studied cousin, Portuguese (Gondar, 1978; Longa, 1994; Carrilho and Sousa, 2010). Given recent renewed interest in the Portuguese inflected infinitive because of the apparent challenges it poses to theories of control (see Quicoli, 1996; Pires, 2001; Modesto, 2010, 2018; Rodrigues and Hornstein, 2013; Landau, 2016; Modesto and Maia, 2017; Barbosa, 2018; Sheehan, 2018a), the Galician inflected infinitive has the potential to be of significant theoretical importance, once its distribution has been clearly established. In this section we review previous descriptive work on the Galician inflected infinitive, drawing extensively on Gondar (1978), the most extensive study to date, before moving on to the arising theoretical issues.

The *Atlas Lingüístico de Galicia* (*ALGa*) (‘Linguistic Atlas of Galicia’), discussed in Gondar (1978) investigated the attestation of the inflected infinitive and its morphological form in the 1970s and detected a certain amount of morphological variation regarding the forms in (1). Although the paradigm in (1) is the dominant one, Gondar notes that some speakers pronounce both the uninflected and inflected infinitive with an epenthetic final -e. (Gondar, 1978, p. 27). More importantly, this -*e* can also appear, for some speakers between the stem and the suffix in the 1^st^/2^nd^ person plural forms giving the alternative forms: *seremos, seredes*. Such forms are, however, reported usually not to be obligatory, but rather alternative variants of the forms in (1) (p. 30). Similarly, Gondar also notes that for some speakers (mainly in A Coruña), there is no distinct plural form for the 2^nd^ person, with the *-es* suffix (2SG) being found also with 2PL subjects. This morphological variation presents an obvious potential challenge for the syntactic investigation of the acceptability of the inflected infinitive: if speakers reject a given example, they might be doing so on purely morphological grounds. As we do not know in advance where which morphological form is used nowadays, it is not possible to adapt the questionnaire examples morphologically and it is obviously not possible to include every possible morphological possibility for each syntactic context as this would lead to a proliferation of examples. This problem can, however, be avoided by using primarily 2sg and 3pl inflections, which are less subject to morphological variation, and this is the approach that we take in our survey.

Gondar (1978, p. 24) notes that partial or full paradigms of the inflected infinitive are found in 136 locations out of 164 in ALGa. The places where the inflected infinitive is not recorded are scattered across the region in all four regions of Galicia (A Coruña, Pontevedra, Ourense, and Lugo), as well as Asturias (which was also included in the Atlas). He speculates that this variation probably has more to do with the “castelanización” (Spanishification) of the people interviewed rather than geography *per se* (pp 25–26), and throughout his study he reiterates his belief that the Galician inflected infinitive is vulnerable due to contact with Spanish. Gondar does report, however, that the full inflectional paradigm is preserved along the coast and in those areas on the border with Portugal, suggesting some geographical effects. In terms of attestation, then, the Galician inflected infinitive can be said to have been widely, though not universally, attested across Galicia in the 1970s.

Gondar is highly critical of previous characterizations of the syntactic distribution of the inflected infinitive. Summarizing several different descriptions (notably those by Saco y Arce, 1967; Carballo Calero, 1974), Gondar notes a number of different syntactic contexts which have been claimed to usually permit and sometimes require inflection. Subject clauses (2) and adverbial clauses (3) are the most frequently discussed contexts, but the complements of verbs with referential subjects are also mentioned (4) (in different descriptive terms by different authors):


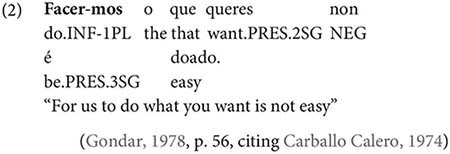



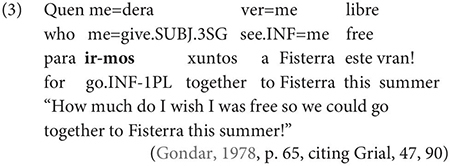



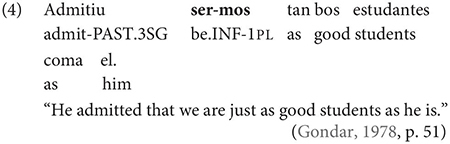


As Gondar notes, the context in (4), while possible in Portuguese and mentioned by Galician grammarians is actually not frequently attested in his corpus search. In such contexts, he notes, where the subject of the embedded clause is not co-referential with the matrix subject, we tend to find a finite subjunctive complement, as would be the case in Spanish (p. 114).^2^

Interestingly, Gondar does note that in contexts which would nowadays be classified as instances of obligatory ‘control’ (in the sense of Landau, 2000), inflected infinitives *are* possible in the complements of verbs. This is true uncontroversially in instances of object control:


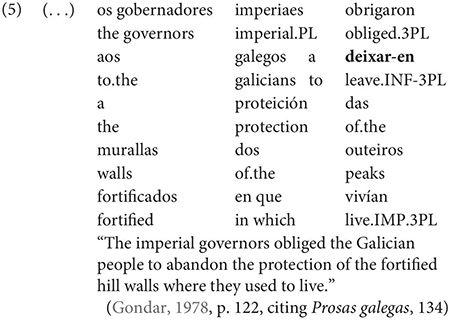


The same is true in European and Brazilian Portuguese (Raposo, 1989; Madeira, 1994; Modesto, 2010; Sheehan, 2018a), though it remains controversial whether such examples are genuine examples of control (see Sheehan, 2018a, b for some evidence they are and Barbosa, 2018 for an opposing view). One control context where European Portuguese speakers generally reject inflection is in instances of what we can descriptively label exhaustive local subject control (see Sheehan, 2018a, but cf. also Fiéis and Madeira, 2017), regardless of whether the matrix clause contains a partial or exhaustive control predicate (in the sense of Landau, 2000). Many Galician grammarians also condemn this usage, especially with restructuring/exhaustive control verbs (see Gondar’s discussion of Saco y Arce, 1967 and Carballo Calero, 1974):


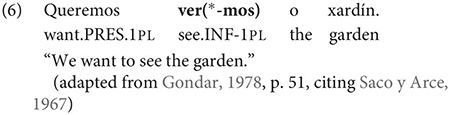



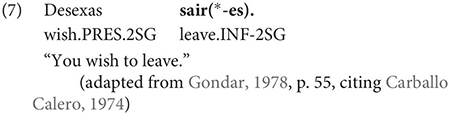


As Gondar notes, however, examples of this kind can be found, even with what would nowadays be called restructuring verbs. Gondar is suspicious of their status, attributing them to over enthusiastic authors with “un desexo de dar á lingua máis forza e vivacidade” (a desire to give the language more strength and vitality):


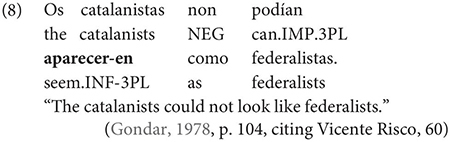


Examples like (9) with partial control matrix verbs are considered less problematic by Gondar but Sheehan et al. (2019) show that they too are proscribed in classroom materials, so must be considered normatively stigmatized:


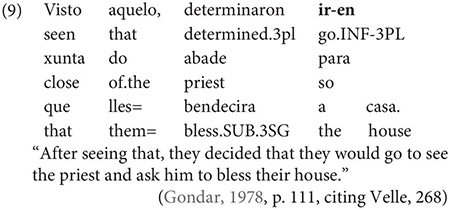


Given that examples like these are also occasionally attested in European Portuguese (Gonçalves et al., 2014), despite native speakers’ judgments, it is an important question how native speakers rate the acceptability of such examples in Galician. Are they part of the grammar of native speakers or artefacts of overenthusiasm, as Gondar claims? In our survey, we limit ourselves to the investigation of partial control verbs, avoiding the complications introduced by restructuring, so that potential contrasts between exhaustive vs. partial control readings can be tested.^3^

One important further context which, Gondar notes, is not discussed by most Galician grammarians is the complement of nouns, in which, he notes, inflected infinitives, preceded by *de* ‘of’ or more rarely *a/p(a)ra* ‘to/for’ are actually very frequent:


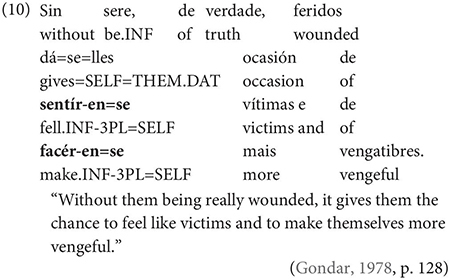


Other contexts, which are not frequent, include the complements of adjectives, comparatives and appositions, as in the following example:


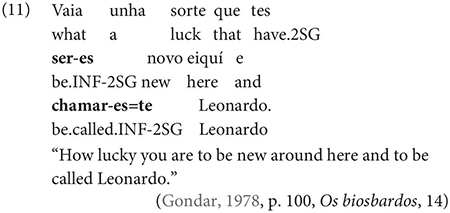


Even in the 1970s, descriptive grammarians report the use of the inflected infinitive in spoken Galician to be in decline. Gondar himself notes that “o que din as gramáticas galegas non-sempre coincide e ás veces mesmo contradice a realidade do uso” (What Galician grammarians say does not always coincide and even sometimes contradicts the reality of use.). Gondar notes that in his oral corpus, especially, the inflected infinitive is very restricted in usage (mirroring the findings reported by Sheehan et al., 2019). In fact, even in his written corpora, the inflected infinitive is still used much less frequently in Galician than in Portuguese, and for many authors it is essentially limited to adverbial clauses (see also Freixeiro Mato, 2002, pp 389–396; Jansegers and Vanderschueren, 2010). Gondar attributes this reduction in use to influence from Spanish, and more specifically, a tendency to use finite complements.

Interestingly, though, Gondar also notes an increase in the use of inflected infinitives in the formal writing of some of his contemporaries attributing it to a desire to “recuperar” (get back) the inflected infinitive and “evitar a súa perda” (avoid its loss) (Gondar, 1978, pp 139–140). Given this observation, an important question is what has happened to the infected infinitive since the 1970s, now that Galician has official language status in Galicia and is widely taught in schools in the region. What are speakers’ intuitions regarding the use of the inflected infinitive in the contexts outlined by Gondar? Is it still limited to adverbial clauses or has its distribution been extended?

#### Theoretical Issues

The distribution of the Galician inflected infinitive is important not only for descriptive and potentially didactic reasons, but also for theoretical reasons. Recent work on Portuguese has highlighted that inflection is often found in contexts which appear to have at least some of the properties of control (Modesto, 2010), and the same appears to be true of Galician. This is potentially problematic for existing theories of control which take the controlled subject position to have a special null case (Chomsky and Lasnik, 1993), to be caseless (Hornstein, 1999), or to be lacking in phi-features (Landau, 2000, 2016). In a theory of grammar in which phi-features on verbs come from agreement with DPs, the implication is that the subject of the inflected infinitive should be a nominative pronoun and hence referential. Indeed, it can be shown that inflected infinitives license overt nominative subjects in both Portuguese and Galician in referential contexts. There are, however, apparently contexts where the subjects of inflected infinitives cannot be free/referential. As Modesto (2010) notes, this poses problems for all existing theories of control.

Sheehan (2018a, b) extends Modesto’s work on Brazilian Portuguese to European Portuguese (and Russian and Icelandic) and proposes a derivational account of these facts whereby the subjects of inflected infinitives begin life as pronouns but, because they are contained in non-finite clauses, are vulnerable to being controlled by thematic heads in the next clause up. In her approach, this is because they move to spec CP in European Portuguese, and she provides evidence for this from (i) clitic placement and (ii) interactions with wh-movement. As Barbosa (2018) notes, however, it is not clear that these examples involve true control (see also Landau, 2016 for a different approach to the Portuguese facts). Sheehan applies the usual diagnostics for control with mixed results (from questionnaire data) and it seems clear that there is substantial variation across speakers, which requires further investigation. Barbosa notes that the main patterns described by Modesto and Sheehan can be explained if these are non-control uses of control predicates, with coerced referential subjects. The main evidence for this comes from (i) the fact that the same verbs which allow ‘control’ with inflected infinitives also permit complements with referential subjects, at least for some speakers (*prometer* ‘promise’ *preferir* ‘prefer’), and from (ii) what Sheehan calls the obviation effect, whereby inflection is banned in instances of exhaustive local subject control. This, both Sheehan and Barbosa note, is the same as the obviation pattern observed with subjunctive clauses with referential subjects (which requires an independent explanation – see Kempchinsky, 2009 for one approach).

The status of Galician is therefore an important part of the non-finite puzzle. Based on Gondar’s description it would appear that, in the complements of verbs, inflected infinitives are *only* possible in instances of control. If true, then the Galician facts do not fall under Barbosa’s proposed analysis for Portuguese. It is therefore important to test this claim empirically: is there a significant difference in acceptability under the same verbs in instances of control vs. non-control? Second, there is reason to believe from attested corpora examples that inflection is even possible in Galician in instances of exhaustive local subject control, though there is clearly variation in this domain and this is clearly stigmatized, as shown by the descriptions of Galician grammarians and in didactic materials. The second important question with respect to control is therefore: is there a significant difference in acceptability of the inflected infinitive in Galician between instances of exhaustive vs. partial control, particularly in instances of exhaustive local subject control? Reliable data on these two issues will enable us to establish (i) whether Galician is really different from Portuguese in this respect and (ii) whether it falls under Barbosa’s proposed analysis of Portuguese.

## Materials and Methods

The study was reviewed and approved by the Ethics Committee of the Faculty of Humanities at Leiden University. Participants read and electronically signed a consent form.

### Materials

We isolated 14 test contexts for inflected infinitives and created multiple examples for each context, ranging between three and five sentences each and giving overall 50 target sentences. The 14 contexts are listed below, with a single example. The contexts were chosen based on corpus examples and the descriptive and prescriptive literature on Galician and Portuguese, in order to make them maximally plausible:

(I) Adjunct clause (5 ex.)


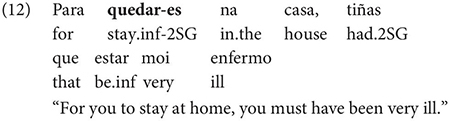


(II) Clausal complement to noun (3 ex.)


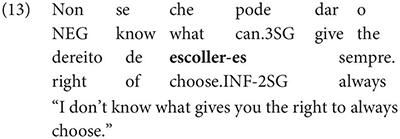


(III) Extraposed clause (3 ex.)


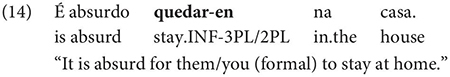


(IV) Factive non-control complemente (3 ex.)


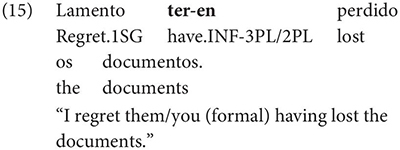


(V) Factive partial control (5 ex.)


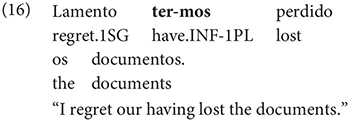


(VI) Epistemic non-control (5 ex.)


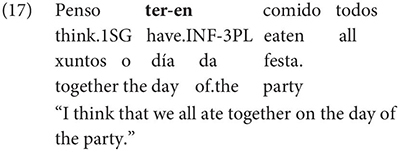


(VII) Epistemic partial control (5 ex.)


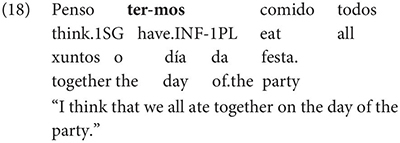


(VIII) Exhaustive object control (3 ex.)


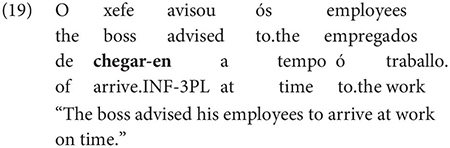


(IX) Partial object control (3 ex.)


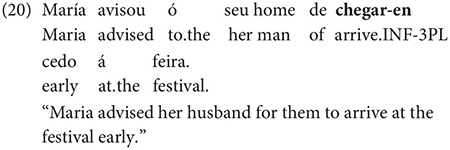


(X) Exhaustive non-local subject control (3 ex.)


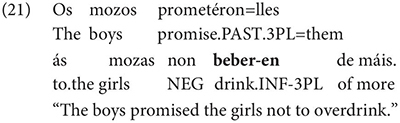


(XI) Partial non-local subject control (3 ex.)


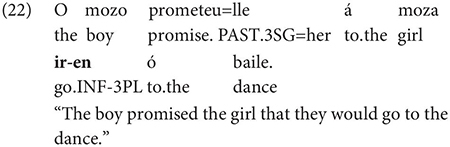


(XII) Exhaustive local subject control (3 ex.)


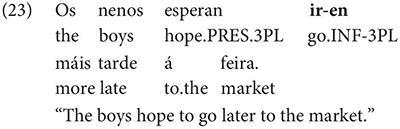


(XIII) Partial local subject control (3 ex.)


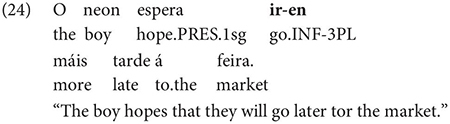


(XIV) Desiderative non-control (3 ex.)


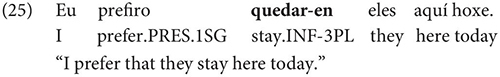


Of these 14 contexts, two groups were minimally contrastive. The first group consisted of two pairs of contexts that each only differed between non and partial control (the two factive contexts IV and V and the two epistemic contexts VI and VII). The second group consists of three pairs of contexts that each only differ in terms of exhaustive and partial control (the two object control contexts, VIII and IX, the two non-local subject control contexts, X and XI, and the two local subject control contexts, XIII and XIV, respectively).

These test items and 24 filler items were recorded as audio-files by a native speaker of Galician (from the Ourense region, not an author of this paper) and embedded in a Qualtrics survey with an additional 15 social profiling questions (placed at the end of the survey). Within the fillers, we included two clearly grammatical items to function as controls (based on the native judgment of one of the co-authors):


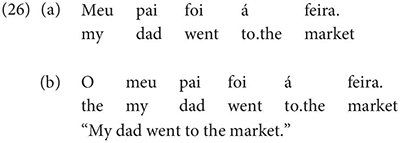


The others fillers were of a more intermediate nature, where variation is expected:


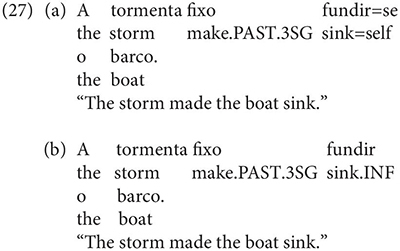


The 74 examples appeared in randomized order and were rated on a five-point scale, using emoticons.^4^

### Procedure

The survey was administered online using Qualtrics. Participants read and electronically signed a consent form. The instructions informed participants that they would hear a series of sentences in Galician and that they were supposed to indicate on a five-point scale (using emoticons) how “acceptable” a sentence was in terms of what they would say to or hear from another Galician-Spanish bilingual speaker. In the scale, a score of 1 stood for “never acceptable” while 5 stood for “always acceptable.” Participants were then presented with the 74 sentences as described above. Each sentence was presented one at a time and the order of presentation was individually randomized for each participant. Participants had to make a choice for each sentence before progressing to the next one and could not return to the previous sentence.

### Participants

A total of 329 participants completed the questionnaire (1053 started but did not finish^5^). Initially, these people were recruited by the researchers’ personal networks but on 2^nd^ November 2017, an article appeared in the Galician language newspaper *Galicia Confidencial*, and this led to large numbers of people filling in the online questionnaire from outside our personal networks.^6^ Most of them were born in administrative regions of Galicia: 178 in A Coruña, 66 Pontevedra, 34 in Lugo, and 28 Ourense. Four others were born in other regions in Spain, 19 outside of these areas. In terms of urban vs. rural place of birth, considering only the participants in the Galician regions, 198 were born in rural areas, 109 in urban areas. Most of the participants, 197, were female, 132 were male. For 249, Galician was the language they learned first (what they considered their mother tongue), for the others, it was Spanish. However, all of them where early bilinguals. The self-reported Galician level was advanced for 226, intermediate for 92 and basic for 11 of the participants.^7^ The age ranged from 16 to 81, with a mean of 36.77 and a median of 38.

Of the 329 participants, 27 people saw shorter versions of the questionnaire not containing eight target sentences that were added later. These examples were added in case the presence of an auxiliary verb might affect grammaticality, but it did not.

Of the 329 participants, we excluded five, two of whom rated all sentences as 5 (fully acceptable), and three of whom rated grammatical controls as either 1 or 2 (unacceptable). This leaves us with 324 participants.

## Results

**FIGURE 1:**
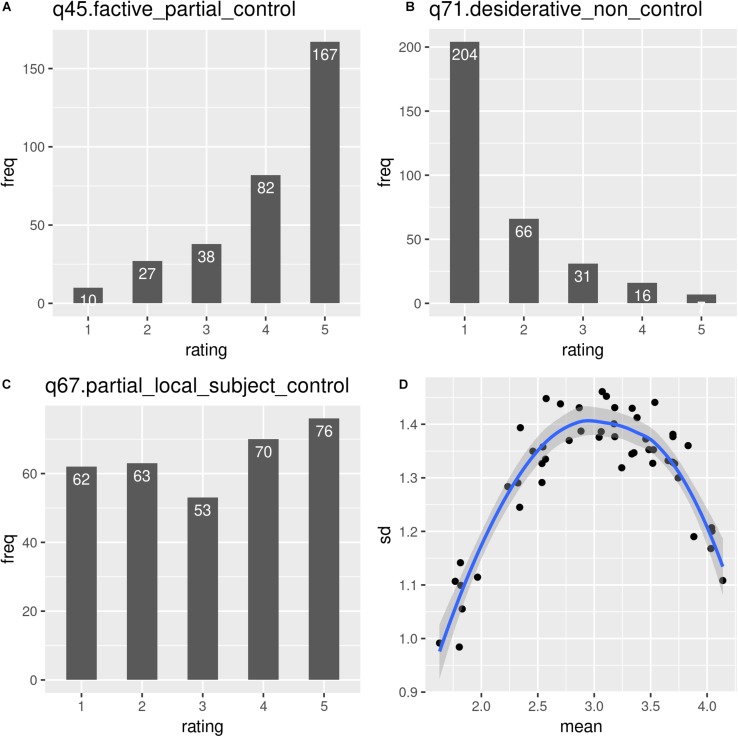
The variation in the ratings across sentences from different contexts. The top left panel **(A)** shows the distribution of the ratings for the sentence that was judged as most grammatical, the top right panel **(B)** shows the distribution of the ratings for sentence which was judged the least grammatical. The bottom left panel **(C)** shows the distribution of a sentence with very high standard deviation. The bottom right panel **(D)** shows the reversed U-shaped curve created by mapping the mean ratings against the standard deviations (graphs were created with gg2plot, Wickham, 2016).

Looking at the raw ratings, we can observe that the ratings come with considerable variation. All 5 emoticons were used for all sentences. In the following, the emoticons are mapped to numbers, with 5 standing for the highest possible rating and 1 for the lowest possible rating. Across all participants we see clear differences in the grammaticality judgments for the individual sentences, ranging from sentence q45, illustrating factive partial control, which was judged as grammatical (4.14), to q71, illustrating desiderative non-control, which was judged as ungrammatical (1.63). The standard deviation for the individual sentences ranges from 0.98 to 1.46. Mapped against the mean ratings, the standard deviations show a reversed U shaped distribution: variation is lower toward the two ends of the scale, with the lowest variation on the lower end, that is, sentences that were judged as the most ungrammatical showed also the lowest variation. Variation was higher when the mean is on the middle of the scale, indicating that judgments on the clear cases are more uniform. Figure 1 illustrates the variation in the ratings across sentences from different contexts by showing (a) the distribution of the ratings for the sentence that was judged as most grammatical (b) the distribution of the ratings for the sentence that was judged the least grammatical (c) the distribution for a sentence with very high standard deviation, and (d) the reversed U-shaped curve (graphs were created with gg2plot, Wickham, 2016).

Turning now to the 14 target contexts, we likewise see clear differences in grammaticality between the individual contexts. Using Cronbach’s α to assess the internal consistency of the sentences making up the contexts, we see again considerable variation, with one context in particular, partial object control, showing little internal consistency while most contexts show solid consistency with values around and above the 0.7 mark. Table 1 gives an overview of the data, showing the contexts in descending order of their mean ratings, and giving the number of sentences making up the context, the mean, the median, the standard deviation and Cronbach’s α for every context.

**TABLE 1:** Overview of the ratings for the 14 contexts, showing the contexts in descending order of their mean ratings, and giving the number of sentences making up the context, the mean, the median, the standard deviation, and Cronbach’s α for every context.

	**No**				
**Context**	**sentences**	**Mean**	**Median**	***SD***	**Alpha**
N complements	3	3.99	4	1.19	0.75
Adjuncts	5	3.72	4	1.37	0.88
Exhaustive non-local subject control	3	3.51	4	1.35	0.76
Factive partial control	5	3.51	4	1.40	0.77
Exhaustive object control	3	3.28	3	1.39	0.72
Partial object control	3	3.26	3	1.43	0.29
Partial non-local subject control	3	3.19	3	1.41	0.73
Extraposed subject	3	3.10	3	1.42	0.68
Exhaustive local subject control	3	2.98	3	1.49	0.61
Epistemic partial control	5	2.76	3	1.41	0.72
Partial local subject control	3	2.74	3	1.39	0.67
Epistemic non-control	5	2.19	2	1.26	0.79
Factive non-control	3	2.09	2	1.21	0.69
Desiderative non-control	3	1.74	1	1.08	0.76

The variation is not surprising: in our crowd-sourced study, we wanted to get a large sample of speakers of Galician from all areas where it is spoken. In addition, we wanted to be able to explore the possible influence of a number of sociological variables on the ratings. That is, it might be that factors such as place of birth, age, or gender influence how the examples are judged. Over and above the inhomogeneity of the target group of our study, people also use Likert scales in different ways, for example differing in their interpretation of the 5 levels of grammaticality in that one consistently uses 4 where another uses 3 to express the same judgments, or two participants exploit the dynamics of the scale in different ways, one using the full range, another just a smaller range (these are well-known issues with Likert scales, see, e.g., Stadthagen-González et al., 2018). And that the contexts themselves show internal variation is also not surprising. While the sentences within a context share the respective syntactic construction, they may differ in many other ways that might influence the overall grammaticality judgment and which we did not control. For example, we did not control for out of context plausibility of the sentences, nor did we control the lexical material and the other grammatical features of the sentences (see also the discussion in Sections 2.1 and 6). Our statistical models in the next section allows us to address most issues coming with this variability, except for the usage of different ranges of the scale; this issue could be addressed by using z-Scores, but this would automatically eliminate any contrasts in the general acceptability ratings, which might in turn be linked to sociolinguistic variation.

### Modeling the Ratings

In our modeling, we use linear mixed effects regression models, in which the participants as well as the sentences occur as random effects. In particular, we will use mixed effects regression models including crossed random effects for participants and sentences (for an introduction to these types of linear mixed effects models, see Baayen et al., 2008). As mentioned above, the potential idiosyncrasies of the participants as well as the individual sentences are addressed in our statistical modeling: both variables are allowed to have random intercepts in the models. For example, over and above any systematic contribution of the predictors, a specific sentence might for reasons not captured by our modeling consistently lead to lower judgments than another sentence. In this case, whatever the model predicts due to the factors in the model is adjusted by a negative number to cater for this idiosyncrasy. The same is true for participants: if, e.g., a participant for idiosyncratic reasons only uses the upper half of the scale, the predictions for this participant are adjusted by a positive number. In other words, the random intercepts capture the tendency of sentences and participants to consistently lead to different values which are not associated with the predictors used in the models.

We were particularly interested in two questions: first, do the 14 different contexts yield grammaticality judgments that are significantly different from grammaticality judgments for clearly grammatical sentences. And if so, do the five minimally contrastive contexts, two of which target the control vs. non-control contrast, and the other three targeting the exhaustive vs. partial control difference, really form different categories. Second, what is the role of the sociological characteristics of the participants in their grammaticality judgments. While the social factors are interesting by themselves, they are also a control for the general reliability of the grammaticality judgments: their inclusion allows us to tease apart the influence of the different grammatical contexts on the ratings from the influence of sociological variables.

In order to have a reference level for the grammaticality judgments, we included the two grammatical fillers, sentence Q22 and Q25, in the data that we modeled. That is, we now use 15 contexts: sentences Q22 and Q25 together as the grammatical reference level, and the 14 target contexts. We first built a model using all 15 contexts. To explore the sociological variables, we included sex, mother tongue, Galician level, whether the place of birth was urban or rural, and age, hypothesizing that these are the most important factors. We started with a model in which all of the sociological variables were allowed to interact. To remove non-significant interactions of predictors as well as non-significant single predictors, we used the step() function from the lmerTest package (Kuznetsova et al., 2017). This function performs automatic backward elimination on random and fixed effects in a linear mixed effects model.

In this first model, each of the 14 contexts predicts a grammaticality judgment that is significantly different from the reference level. Of the sociological factors, Galician level and age play no role, while place of birth (urban vs. rural), mother tongue (Galician vs. Spanish), and gender participated in a three-way interaction^8^.

We then considered whether the two minimally contrastive contexts are associated with distinct grammaticality judgments. The first context consisted of the two pairs contrasting non-control and partial control: (i) epistemic non-control vs. epistemic partial control and (ii) factive non-control vs. factive partial control. The second context targeting the contrast between exhaustive and partial control consisted of three pairs: (i) exhaustive local subject control vs. partial local subject control, (ii) exhaustive non-local subject control vs. partial non-local subject control, and (iii) exhaustive object control vs. partial object control. To test for a difference between these pairs, the first model was compared to models in which the respective pair of contexts was conflated into one context, so that there were only 13 different target contexts. ANOVAs were then used for model comparison. When the model with the distinct contexts was not significantly better than the model with the corresponding pair conflated, this was taken to indicate that the minimal contrast did not play a role in arriving at the grammaticality judgments. This procedure revealed that non-control contrasts with partial control: collapsing epistemic non-control with epistemic partial control led to a significantly worse model than keeping the two contexts separate. Likewise, collapsing factive non-control with factive partial control led to a significantly worse model.

In contrast, the difference between exhaustive and partial control played no role in grammaticality judgments. For each of the three pairs, there was no significant difference between a model that collapsed the two contexts of a pair and the model that kept them apart, making the sparser models, that is, those with the collapsed contexts, the preferable models. In the final model, these six contexts were consequently conflated into just three contexts, local subject control, non-local subject control, and object control. Note that, incidentally, conflating the two contexts of partial and exhaustive object control into one also made the resulting larger context more consistent, leading to a Cronbach’s α of 0.71.

The final model is presented in Table 2.

**TABLE 2:** Final mixed effects model for sentence grammaticality.

**Random effects**					
**Groups**		**Name**	**Variance**	***SD***	

Participants		(intercept)	0.4709	0.6862	
Sentences		(intercept)	0.1406	0.3750	
Residual			1.1543	1.0744	
15496, groups: participants, 302; sentences, 52					

**Fixed effects**					

	**Estimate**	***SE***	**df**	***t*-value**	**pr(>|t|)**

(Intercept)	4.599933	0.276989	45.07	16.607	<2 E-016
Adjunct	–1.007494	0.318042	39.99	–3.168	0.002940
Complement of N	–0.729029	0.346961	39.97	–2.101	0.041977
Desiderative non-control	–2.980684	0.346961	39.97	–8.591	1.28e−10
Epistemic non-control	–2.557176	0.318042	39.99	–8.04	6.98e-10
Epistemic partial control	–1.970977	0.318042	39.99	–6.197	2.50e-07
Extraposed subject	–1.634106	0.346961	39.97	–4.71	2.98e-05
Factive non-control	–2.660727	0.347020	39.99	–7.667	2.25e-09
Factive partial control	–1.218349	0.318018	39.98	–3.831	0.000441
Local subject control	–1.869757	0.310331	39.97	–6.025	4.37e-07
Non-local subject control	–1.376380	0.310331	39.97	–4.435	7.02e-05
Object control	–1.460265	0.310331	39.97	–4.706	3.02e-05
Sex	0.149900	0.113706	293.98	1.318	0.188424
Mother tongue	–0.155191	0.188275	294.77	–0.824	0.410449
Birth place	0.168013	0.139700	293.97	1203	0.230074
Sex:mother tongue	–0.001562	0.297715	294.16	0.005	0.995819
Sex:birthplace	–0.197195	0.205898	294.07	–0.958	0.338984
Mother tongue: birthplace	–0.075094	0.276370	294.50	–0.272	0.786032
Sex:mother tongue:birthplace	0.818804	0.408080	294.25	2.006	0.045720

Marginal *R*^2^ = 0.2262928, conditional *R*^2^ = 0.4942296

*The top section shows the random effects: the model includes random intercepts for participants and sentences. The bottom section shows the fixed effects. First, it shows the estimates associated with the different contexts, then the influence of the sociological factors. The sociological predictors participate in a three-way interaction.*

The top section of Table 2 shows the random effects: the model includes random intercepts for participants and sentences. The bottom section of Table 2 shows the fixed effects, that is, those predictors that are associated with differences in the grammaticality judgments. First, it shows the estimates associated with the different contexts, then the influence of the sociological factors. The sociological predictors participate in a three-way interaction. Note that intercept of the model, 4.60, is the value on the Likert scale that the model predicts for a female native speaker of Galician who was born in a rural area for the two sentences making up the fully grammatical context. The estimates of the other contexts are therefore deviations from this level of grammaticality. For example, the model predicts that a speaker with the same sociological characteristics will rate a sentence where the infinitive occurs as a clausal complement of a noun as 3.87, that is, the intercept, 4.60, minus the estimate for the context, 0.73.

The *R*^2^ values at the bottom of the table show the variance explained by the model. The marginal *R*^2^ values give the variance explained by the fixed factors, and the conditional *R*^2^ values represent the variance explained by the whole model, that is, including the random effects. Marginal and conditional *R*^2^ values were calculated with the r.squaredGLMM() function in the MuMIn package (Bartoń, 2016), an implementation which is in turn based on R code from Nakagawa and Schielzeth (2013) and Johnson (2014).

**FIGURE 2:**
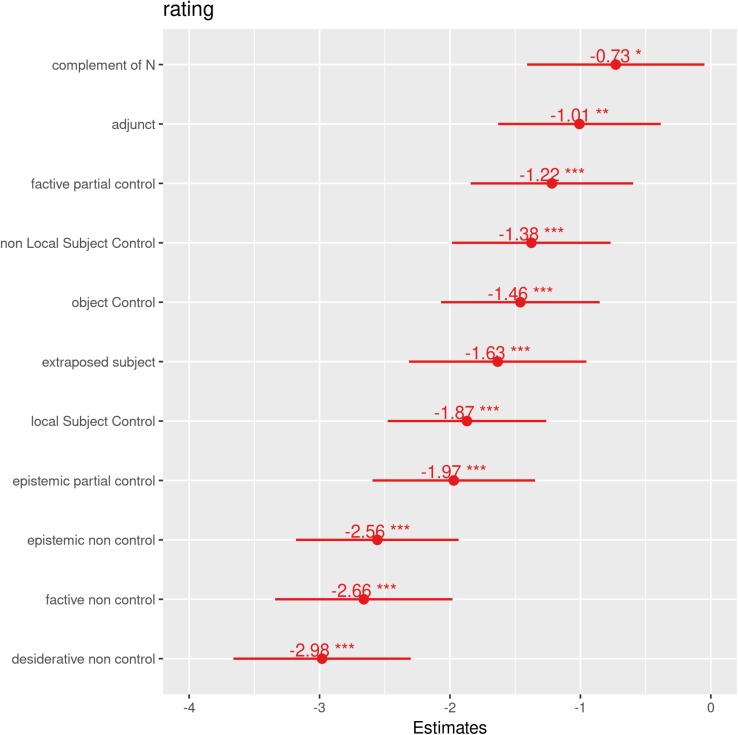
The fixed effects in the final model that do not participate in the three-way interaction, ordered by the estimates (the figure was produced using the sjPlot package, Lüdecke, 2018).

The fixed effects not participating in the interaction are visualized in Figure 2, ordered by the estimates (the figure was produced using the sjPlot package, Lüdecke, 2018).

Reassuringly, the relative order of the contexts corresponds to the order of the means of their raw ratings, except, as explained above, the contexts only differentiated by the contrast between exhaustive and partial control have been collapsed into three combined contexts, because there was no significant difference in judgments tied to this contrast.

**FIGURE 3:**
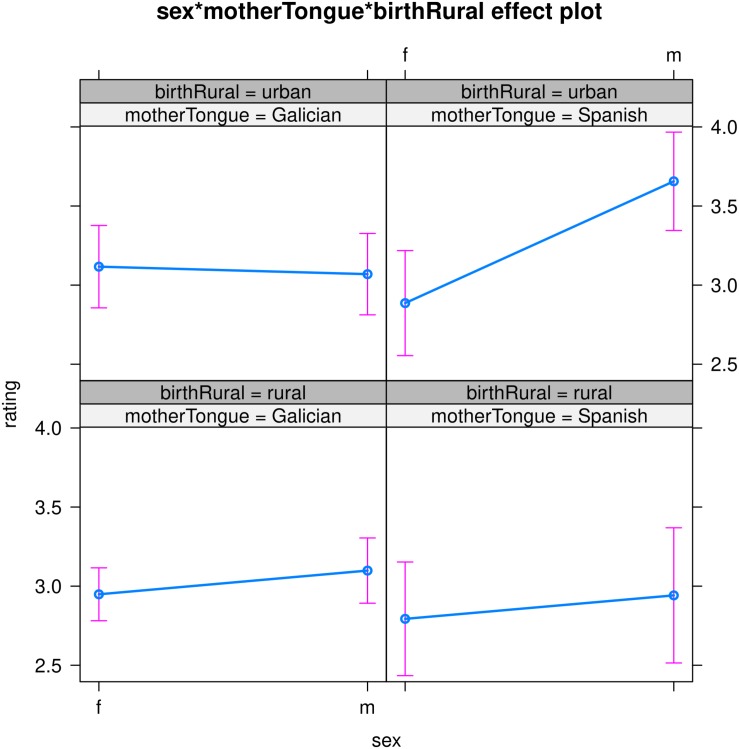
The three-way interaction between gender, mother tongue and place of birth in the final model (the figure was produced using the effects package, Fox, 2003).

The three-way interaction between gender, mother tongue and place of birth is visualized in Figure 3, using the effects package (Fox, 2003).

The top two panels show the interaction mother tongue and gender for the urban population, the two bottom panels show the interaction between the two for the rural population. Interestingly, the only constellation where there is a clear difference in judgments (with non-overlapping confidence intervals) is for the urban speakers with Spanish as their mother tongue. Here, males were more accepting than females in their grammaticality judgments. In order to check whether this effect was associated especially with the inflected infinitives, we also modeled the filler items with the same sociological variables, and we obtained the same three-way interaction. That is, this three-way interaction is stable across all our data and seems to obtain for all Galician data. In short, this group of urban-born male bilinguals with Spanish as a mother tongue were more accepting in general across all sentences, including fillers.

Note that while the sociological variables via this three-way interaction give rise to a model that is significantly better than a model containing just the different contexts, model comparison shows that the grammatical contexts account for the larger amount of variation in the data. The grammatical contexts by themselves explain 20 percent of the variation in the data, while the sociological variables only add another 2 percentage points.

### Implications for Syntactic Theory

The results of the questionnaire call into the question the idea that the inflected infinitive is in decline in present day Galician. While there is a great deal of variation, there is clearly a shared set of contexts which permit inflected infinitives for the vast majority of speakers as well as contexts which do not, with little variation across speakers in these contexts. The general picture which emerges is that inflected infinitives with a referential subject are possible in strong islands (adjunct clauses and complements of nouns), but not in non-islands (complements of verbs). This contrasts with the patterns reported for Portuguese and older varieties of Galician in which examples of the second kind are clearly grammatical. This cannot be handled merely as a matter of selection. The results of the survey show that verbs (*lamentar* ‘regret,’ *odiar* ‘hate,’ *pensar* ‘think,’ and *afirmar* ‘confirm,’ etc.) *can* select a clausal complement containing an inflected infinitive, but only in instances of control. Though these examples are less acceptable than the core examples just mentioned, and subject to more interspeaker and intraspeaker variation, they are significantly more acceptable than examples without control. Where the subject of the embedded clause has a distinct referent from the matrix subject, the inflected infinitive is much more systematically rejected and, presumably, a finite complement is required (as Gondar, 1978 notes). It follows then that these apparent instances of control cannot involve accidental co-reference, as Barbosa proposes for Portuguese. These verbs allow inflected infinitives *only* in instances of control and not elsewhere (though slightly more marginally, and not for all speakers), a pattern attested also for at least some speakers of European Portuguese, where again, there is substantial variation across speakers (Sheehan, 2018a).

So how can we account for the fact that inflected infinitives with referential subjects are limited to strong islands in Galician? We propose that an account can be given in terms of phases. Gondar actually makes the point that what regulates the availability of inflected infinitives with referential subjects is “[a] unión menos estreita do infinitivo co verbo principal” (the less narrow union of the infinitive with the main verb) (Gondar, 1978, p. 127). In other words, in order to have a referential subject, a non-finite clause needs to be in a distinct domain from the verb in the next clause up. This intuition, which is strongly empirically supported by our survey, is easy to formulate in terms of phase theory. According to Chomsky’s (2001) Phase Impenetrability Condition 2 (PIC 2), the complement of a phase head is transferred when the next highest phase head is merged. Taking the clausal phase heads to be C and voice in this case (see Sheehan and Cyrino, 2017 for independent justification based on work by Harwood, 2015 and many others), it follows that where a non-finite CP is embedded directly under a verb, the inflectional domain (IP) of the embedded clause remains visible to the thematic domain of the higher clause (vP). This is illustrated in (28): vP has been transferred, upon merger of C, but IP is still visible to the higher v because the next highest phase head (voice) is yet to be merged:


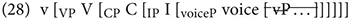


If one formulates the control relation in terms of syntactic Agree (Landau, 2000; Gallego, 2011; McFadden and Sundaresan, 2018), then phase theory leads us to expect that it will be available here as I and v are in a local domain. Note that this is true whether the controlled subject is a covert pronoun in spec IP or the pronominal inflection in I (see Barbosa, 1995; Alexiadou and Anagnostopoulou, 1998; Sheehan, 2016 for discussion of this issue, and further references). All that needs to be said to explain the Galician pattern is that control is obligatory in such contexts. Because I is accessible matrix v and embedded I have to form a thematic dependency. This is why inflected and uninflected infinitives embedded under verbs are always controlled^9^.

Crucially, this control relation is not possible where the CP is not embedded under v but rather occupies a strong island position. Where clauses function as adverbs, there is no clear consensus as to how this should be analyzed structurally, but it is clear that the result is an opaque domain which is not accessible to the main clause, as can be seen by the impossibility of wh-extraction:


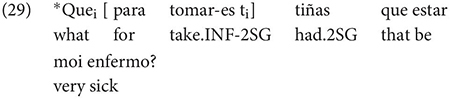


Adopting the proposal of this effect in Uriagereka (1999) and Nunes and Uriagereka (2000), we assume that the strong islandhood of adjuncts results from the fact that they are atomised prior to being merged with the main clause. This renders them opaque to syntactic probing, explaining why control is not possible.^10^ Where a non-finite CP is embedded under a noun, it is probable that the obligatory intervening presence of P is crucial. If P is also a phase head, then when it is merged, the complement of C (IP) will be spelled out, rendering the inflectional domain of the lower clause invisible to thematic probing by a higher v, as illustrated here:


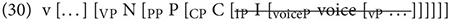


This explains, again, the fact that the complements of N are also strong islands for wh-extraction:


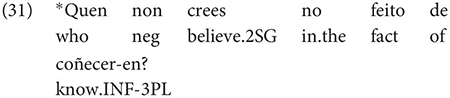


In fact, although we could not test the status of this claim in our survey, it has been noted that referential subjects are more acceptable under verbs in Galician where a preposition intervenes between the verb and its clausal complement (see Gondar, 1978, p. 51), as in (32). Under our analysis, this follows straightforwardly if P is a phase head, rendering the lower IP invisible to the higher thematic domain (vP).


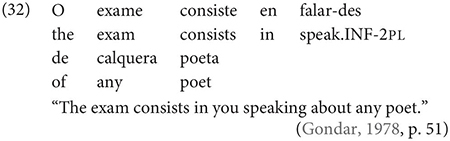


The distribution of the inflected infinitive is therefore of potential central importance to our understanding of core theoretical issues such as finiteness, and how that can be analyzed in terms of phases. As pointed out by a reviewer, the Galician facts provide further support for a scalar view of finiteness as these are forms which despite inflection have certain non-finite properties (see Ledgeway, 1998; Landau, 2004).

There are two remaining questions arising from our data. First, how can we explain the behavior of extraposed subject clauses and, second, how can we explain the lack of any obviation effect in Galician. Let us first consider extraposed subject clauses. While this is one of the core contexts described for the Galician inflected infinitive by traditional grammarians, with examples of this kind also being fairly frequently found in didactic materials (Sheehan et al., 2019), these examples were subject to substantial inter- and intra-speaker variation in our survey, patterning much more similarly to the more marginal control contexts than to the core acceptable contexts (adjunct, complement to N) and being less acceptable overall even than object control and non-local subject control. Can our proposed analysis account for this? Extraposed subject clauses are usually claimed to behave like weak islands (see Cinque, 1990), allowing extraction of some phrases, sensitive to complex semantic factors (Pesetsky, 1987; Cinque, 1990; Starke, 2001; Sabel, 2002; Szabolcsi, 2006). In fact, recent approaches suggest that the ultimate explanation for weak islandhood must be semantic, given the extent of these complexities (Abrusán, 2007, 2011). If this is the case then, in syntactic terms, wh-extraction is possible and so extraposed clauses do not constitute syntactic islands. This means that, according to our proposal, they ought to be controllable where they are c-commanded by a thematic head. This is where things get murkier, though. If we assume that extraposed subject clauses are externally merged in a subject position, then, according to Uriagereka (1999) and Nunes and Uriagereka (2000), they would be atomized and hence behave as strong islands. If, on the other hand, they are base generated in a complement position, then they would be visible to the thematic adjective/verb which selects them and this would be sufficient to rule out the possibility of a referential subject. Our account thus allows for the variability in this domain.

The final puzzle we are left with is the lack of the obviation effect in Galician, so that inflected infinitives are acceptable (for some speakers) even in cases of exhaustive local subject control, unlike in Portuguese. In actual fact, Galician behaves as expected, in this respect, if these are genuine instances of control then it is European Portuguese (and Russian and Icelandic, which disallow ‘cased’ control in this same context) which behave unexpectedly (see Sheehan, 2018b). We therefore leave this puzzle to future research, noting only that the lack of obviation effects in this context in Galician makes all the more improbable an accidental co-reference analysis like that proposed by Barbosa (2018) for Portuguese. As both reviewers point out, an important area for future research is to extend our analysis to (European and Brazilian) Portuguese, but as this depends partly on contested empirical generalizations regarding Portuguese, this must be preceded by sociolinguistically grounded research on Portuguese inflected infinitives.

### Implications for Crowdsourcing

Crowdsourcing allows researchers to develop and test hypotheses with many naïve speakers within days at relatively low cost. This enables the recruitment of more diverse and representative participants than in many lab settings. It has also been shown to provide results that are as reliable as lab-based experiments (see Erlewine and Kotek, 2016). In fact, our survey included a wide range of social profiling questions so that we were able to statistically test the influence of sociological variation on the judgments, something which is often not controlled for in syntactic work. As detailed in the results section, in our analysis we focussed on a set of core social variables and found that only the interaction of place of birth (urban vs. rural), mother tongue (Galician vs. Spanish), and gender were significant factors in our model, while age and self-reported Galician level played no role. The fact that these social variables by themselves account only for 2% of the variation is further corroboration of the reliability of crowdsourcing. Including so many sociological profiling questions also makes our dataset more valuable for other researchers wanting to explore our data further: any of the variables or combinations thereof can be tested, and while even in our large dataset combinations soon become unique, the data might nevertheless show interesting trends that can be used in the design of further studies. Moreover, where syntactic phenomena are of a gradient nature, as in the case of the Galician inflected infinitive, data collection should include a quantitative component (see Wasow, 2009 on gradiency in grammars). This is precisely what crowdsourcing allows us to do, enabling us to provide a more empirically robust picture of the acceptability of inflected infinitives by native speakers.

This method raises important questions regarding the relationship between I-language and E-language, in the sense of Chomsky (1986). Generative grammarians are traditionally concerned with understanding the working of I-languages, the internal grammars of individual speakers and of using these to study the nature of Universal Grammar. E-languages, such as French or English, in as much as they exist, are the external product of a collection of I-languages which while being largely consistent, may be subject to considerable low-level differences. There is a question, then, regarding the status of data from crowdsourcing in this juxtaposition. Undeniably, this data differs from corpus data in that it stems from individual intuitions rather than production. Moreover, at the extremes of acceptability and unacceptability where there is little interspeaker variation, we can assume that speakers in a speech community share this aspect of grammar in their respective I-languages. At the middle of the scale, where there is considerable inter- and even intraspeaker variation, the relationship to I-language is less clear cut. It is likely that at least some of this variation must reduce to differences between individual I-languages. This variation also seems to point at a gradient notion of grammaticality, however, the existence of which is widely acknowledged but not accommodated in mainstream theoretical approaches (with Optimality Theory being a notable exception).

Our results are somewhat surprising, given that inflected infinitives have been argued to be extremely restricted in spoken Galician, production data would help so that we could see not just what inflected infinitive forms are possible in Galician, but also what their relative frequencies are (cf. Wasow, 2002; Bresnan, 2006). However, the unavailability of a corpus of contemporary oral Galician makes it unfeasible to identify the probability of the inflected infinitive in a particular context, and in any case, as Gondar (1978) notes, where there is optionality, there are many contributing factors, such as distance between verbs, style and not least degree of ‘Spanishification.’

The status of Galician as a minority language with a history of oppression raises certain special ethical and methodological issues. While acceptability judgment tasks like the one we conducted provide valuable data on speakers’ linguistic intuitions, they can also become entangled with prescriptivist views on language. Native speakers of Galician are not necessarily familiar with the prescriptive norm, to the extent that one exists for this grammatical phenomenon, which can make them feel insecure about their own language. This self-doubt can have an effect in their judgments (for example they may tend to stay in the middle of the scale) or even prevent potential participants from wanting to participate in any language-related study. At the same time, since the norm is unclear regarding the contexts where inflected infinitives can be used, this may also lead to a more intuitive response, unaffected by prescriptive pressures.

It is difficult to avoid problems such as these, but the use of an audio-questionnaire may have helped to mitigate some of these effects as it is well-known that attitudes to spoken languages are much less affected by the aforementioned issues (Koronkiewicz and Ebert, 2018), though this did have some drawbacks. The recordings were necessarily made by a speaker from one specific region (Ourense). This introduced a further set of potential confounds related to regional pronunciation. This factor does not arise, of course, in written questionnaires. An alternative would have been to use synthesized speech, striving for a sociolinguistically neutral version.

Despite the advantages of technology, it also brings its own issues. A major issue with the use of technology is that it immediately biases who is able to participate in a study. In the case of our study, the use of audio clips created even more substantial barriers to participation as high-speed broadband was required in order to listen to stimuli and this is simply not available in all rural communities in Galicia. Given that urban/rural birth was a significant sociological factor determining the use of inflected infinitives, these concerns need to be born in mind by linguists, as we run the risk of describing urban vernaculars and rendering rural variants invisible. Where gender is also a relevant factor, as again it is here, there is a risk that skewings can arise as females are more likely to fill in online questionnaires (Smith, 2008). Educational levels (which normally entail familiarity with Galician norms) are also factors to consider.

Finally, we would like to mention issues of participant recruitment and echo-chamber effects. Unfunded crowdsourced research necessarily relies on the voluntary contributions of participants. The use of personal networks can be problematic in such cases, creating a potential echo-chamber effect whereby participants provide the data that they think you would like to receive. Our survey allows us to test the existence of such an effect because large numbers of participants in our survey came from outside our personal networks. Interestingly, statistical analysis shows no significant difference between the data collected before and after the publication of the article in *Galicia Confidencial* (2^nd^ November, 2017) which led to the wider distribution of the survey link. This suggests that, even when they are not economically rewarded, both contacts and unknown participants can be trusted to provide data on minority languages honestly. In total only five people were eliminated from the study, about 1.5% of total participants.

## Conclusion

The use of online questionnaires of this kind enables researchers to collect large amounts of data and to control for a host of sociological factors which might otherwise be skewing our description of syntactic phenomena. Crowdsourcing of this kind also allows us to eliminate noise from results, leaving us with a consensus view of clearly acceptable/unacceptable phenomena and giving us a better handle on variable phenomena, which are usually described as ‘?/??’ in linguistic descriptions. Such surveys are particularly useful and important in relation to the last kind of phenomena, which are often left out of theoretical discussions, or sidelined. In the case of the Galician inflected infinitive, this first large-scale survey shows that speakers systematically allow inflected infinitives with referential subjects in strong islands and fairly systematically reject them in non-islands. It also shows that inflected infinitives can appear in instances of what looks like control, posing potential problems for approaches to control in which the controlled subject is underspecified, lacking inherent phi-features. We have proposed an analysis of this distribution, based on phase theory, whereby the subjects of non-finite clauses are susceptible to control, regardless of their inflectional properties. Finally, we have shown that acceptability is subject to sociolinguistic variation, by gender, urban/rural birthplace and declared dominant language. Further explorations of our existing dataset may isolate other relevant sociolinguist interactions.

## Data Availability

The dataset analyzed in this study is published as Parafita Couto et al. (2019).

## Ethics Statement

The experiment followed the Ethics Code for linguistic research in the faculty of Humanities at Leiden University, which approved its implementation.

## Author Contributions

MSh and MP conceived and designed the study. MSc conducted the statistical analysis and MSh was responsible for the theoretical interpretation of the data. MSh drafted the manuscript. All authors wrote sections of the manuscript, contributed to manuscript revision, and read and approved the submitted version.

## Conflict of Interest Statement

The authors declare that the research was conducted in the absence of any commercial or financial relationships that could be construed as a potential conflict of interest.

## Footnotes

2 Gondar (1978) also notes that, unlike Portuguese, Galician did not extend the use of the inflected infinitive to the complements of causative/perception verbs, and in this sense can be considered conservative compared with Portuguese (p. 121). As he notes, this conservatism in not surprising once we consider that inflected infinitives with referential subjects are rare anyway in the complements of verbs.

3 Sheehan et al. (2019) also checked for examples of inflected infinitives in naturalistic conversations in the *Corpus Oral Informatizado da Lingua Galega (CORILGA)* (Digitised Oral Corpus of the Galician Language) (García-Mateo et al., 2014) which consists of 98 h of informal and formal conversations recorded from 1960 onwards. They report only 71 examples of inflected infinitives in the entire corpus, found in the following contexts: adjuncts (*n* = 44, 62%), complements of nouns (*n* = 10, 14%), complements of Adj (*n* = 3, 4%); extraposed subject clauses (*n* = 2, 3%) and, most notably in exhaustive local subject control contexts (*n* = 3, 4%). The attestation of these kinds of examples suggests that, for some speakers at least, they might be acceptable, though the numbers involved are obviously very small.

4 https://www.qualtrics.com

5 Some participants reported being unable to finish it due to poor internet connection or signal in their cell phones. Internet access is still not widespread in Galicia, particularly in rural areas. See: https://www.farodevigo.es/sociedad/2017/10/06/galicia-comunidad-hogares-disponen-conexion/1762310.html We discuss technical challenges such as this in section 6.

6 http://www.galiciaconfidencial.com/noticia/66048-usamos-galegos-infinitivo-conxugado?fbclid~=~IwAR3FroMUXhKXEKq1W3cauzgBTmMtaMSblhPhkyj-4raNzP01ow66ofxlGB8

7 A reviewer points out that self-report is not always a reliable way to assess proficiency level and we would agree. However, in the context of an online survey, other, more objective measures are obviously ruled out. In such cases, it is common practice to rely on “the capacity of the individuals to self-report accurately, a roughly equivalent sense among individuals of what self-report means and an unbiased willingness to communicate their proficiency levels” (Deuchar et al., 2014, p. 101).

8 An anonymous reviewer is surprised, as we were, that age is not a relevant factor. This could be an affect of sampling. While the age span of our participants ranges from 16 to 81, the values of the first and third quartiles are 28 and 44, respectively. That is, half of the participants come from the 28 to 44 age range. Another possible reason could be the particular situation of Galician as language which is subject to revitalization efforts.

9 One might ask at this point why we do not generally find control into finite clauses in Galician. We do, of course, in some languages (see Landau, 2004, 2015), but in these cases there is reason to believe that controlled finite clauses are smaller than uncontrolled ones. In fact, the very notion of finiteness has been shown to be deeply problematic for this reason. To explain the Galician (and English) pattern, we can hypothesise that what distinguishes controllable (non-finite) from uncontrollable (finite) is that the latter are headed by two phase heads. This is a plausible explanation because independent evidence suggests at least two heads in the C-domain (Force and Fin) (see Rizzi, 1997). If there are two C-related phase heads in finite clauses in Galician (and English), then the inflectional domain of a lower clause (IP) will no longer be visible to the thematic domain of the next clause up (vP). See McFadden and Sundaresan, 2018 for a approach which has some parallels with this proposal.

10 A reviewer points out that we have not actually shown that control into adjuncts is not possible. We are implicitly assuming complementarity of referential subjects and control, so that if a context permits a referential subject, then it is not a control context. Co-reference between a main clause argument and the subject of an inflected infinitive in an adjunct position is possible, but we assume that this is not true control (see Sheehan, 2018a on European Portuguese). We agree with the reviewer that it would be useful to test this implicit assumption via the usual diagnostics (see Hornstein, 1999; Landau, 2000). The same reviewer helpfully highlights contexts where nominal complements seem to require control and disallow referential subjects: (i) Os americanos foram os primeiros a (^*^os britânicos) pisarem na lua. [Brazilian Portuguese] the Americans were the first to (the British) step.inf.3pl on.the moon. Such examples involve prepositional infinitives, which have long been known to present specific challenges for syntactic theory (see Raposo, 1989). While we agree that it would interesting to investigate such contexts in Galician, we leave this as a matter for future research.
